# Clinical characteristics of *Chlamydia psittaci* pneumonia diagnosed by next-generation sequencing: An observational study

**DOI:** 10.1097/MD.0000000000050850

**Published:** 2026-09-25

**Authors:** Rongchun Wang, Na Li, Jia Tian, Xiangbo Zeng

**Affiliations:** a Department of Pulmonary and Critical Care Medicine, The Second People’s Hospital of Hunan Province (Brain Hospital of Hunan Province), Changsha, Hunan, China.

**Keywords:** bronchoalveolar lavage fluid, *Chlamydia psittaci* pneumonia, clinical features, next-generation sequencing

## Abstract

*Chlamydia psittaci* pneumonia is often underdiagnosed due to nonspecific presentation and limited diagnostic tools, with scarce data on the comparative utility of metagenomic next-generation sequencing (mNGS) versus targeted next-generation sequencing (tNGS) in this setting. This study aimed to characterize the clinical features of *C psittaci* pneumonia and provide comparative data on mNGS and tNGS, with emphasis on diagnostic accessibility, while also quantifying underreported features such as hyponatremia. A retrospective analysis was conducted on 10 patients with *C Psittaci* pneumonia admitted to the Department of Respiratory and Critical Care Medicine at the Second People’s Hospital of Hunan Province between January 2022 and February 2025. Clinical data, laboratory tests, imaging findings, treatment regimens, and outcomes were collected and analyzed descriptively. Among the 10 patients (6 male, 4 female; median age 70 years), 9 had underlying comorbidities and 2 had severe pneumonia. A history of avian exposure was reported in 40% of cases. All patients presented with fever (mean temperature: 39.5 ± 0.7°C), accompanied by nonspecific symptoms including cough (n = 4), chills (n = 5), anorexia (n = 5), and myalgia (n = 3). Laboratory findings revealed normal white blood cell counts in 8 cases, while lymphocytopenia, elevated inflammatory markers, abnormal liver enzymes, and hyponatremia were observed in 9 patients. Imaging predominantly showed unilateral lung consolidation (n = 5) or ground-glass opacities (n = 3), with pleural involvement in 9 cases. mNGS and tNGS were performed on bronchoalveolar lavage fluid in 5 cases each; tNGS provides a more cost-saving and rapid alternative. Post-diagnosis, treatment with moxifloxacin, doxycycline, or omadacycline for 10 to 21 days yielded generally favorable outcomes. *C psittaci* pneumonia is characterized by high fever and systemic symptoms, with imaging typically showing unilateral consolidation, ground-glass opacities, or mass-like lesions with pleural involvement, accompanied by liver injury, elevated inflammatory markers, and hyponatremia. Both tNGS and mNGS are effective diagnostic tools, and tNGS provides a more cost-saving and rapid alternative. Early treatment with fluoroquinolones or tetracyclines confers significant therapeutic benefits.

## 1. Introduction

*Chlamydia psittaci* is a zoonotic, obligate intracellular pathogen capable of infecting at least 465 bird species, particularly those in the Psittacidae (parrot) and Columbiformes (pigeon/dove) orders. In daily life, pigeons and pet parrots are common reservoirs. Transmission primarily occurs through inhalation of aerosols contaminated with secretions from infected animals or asymptomatic carriers.^[1,2]^ A 2017 meta-analysis indicated that *C psittaci* accounts for approximately 1% of community-acquired pneumonia (CAP) etiologies, though significant variation exists between studies, ranging from 0 to 6.7%.^[3]^ A prospective multicenter study on CAP identified *C psittaci* as an important emerging pathogen in severe CAP (SCAP).^[4]^

Diagnosis of *C psittaci* pneumonia is often challenging due to nonspecific symptoms and limitations of traditional diagnostic methods.^[5]^ These diagnostic constraints also contribute to insufficient clinical awareness of the disease.^[6]^ Next-generation sequencing (NGS) has revolutionized the diagnosis of infectious diseases. NGS primarily includes metagenomic NGS (mNGS) and targeted NGS (tNGS). While mNGS demonstrates excellent performance in diagnosing *C psittaci* pneumonia, the cost-effectiveness advantage of tNGS is gaining attention.

Previous studies^[2,7–11]^ have described the clinical features of *C psittaci* pneumonia diagnosed by NGS, but several knowledge gaps remain: no direct comparison of mNGS versus tNGS in psittacosis; hyponatremia prevalence reported only anecdotally; pleural involvement and mass-like lesions not systematically quantified on computed tomography (CT); and real-world treatment failure pathways with empirical β-lactams poorly documented. The present study addresses these gaps by providing comparative data on cost and turnaround time for mNGS versus tNGS, quantifying the prevalence of hyponatremia and pleural involvement, and documenting the complete treatment trajectory from ineffective β-lactams to successful tetracycline/fluoroquinolone switch. This study retrospectively analyzed the clinical data, imaging features, treatment courses, and outcomes of 10 patients with *C psittaci* pneumonia confirmed by NGS, aiming to enhance clinicians’ understanding and management of this disease.

## 2. Materials and methods

### 2.1. Study subjects

This retrospective study analyzed 10 cases of *C psittaci* pneumonia admitted to the Department of Respiratory and Critical Care Medicine at Hunan Second Provincial People’s Hospital between January 2022 and February 2025.

Inclusion criteria: met diagnostic criteria for pneumonia; detection of *C psittaci* nucleic acid sequences in bronchoalveolar lavage fluid (BALF) by either mNGS or tNGS. Exclusion citeria: incomplete radiological data.

Patients were classified as having severe psittacosis pneumonia if they met diagnostic criteria for SCAP,^[12]^ otherwise as non-severe psittacosis pneumonia. SCAP was defined as meeting any one major criterion: requiring endotracheal intubation and mechanical ventilation; septic shock requiring vasopressors after adequate fluid resuscitation; or meeting 3 or more minor criteria: respiratory rate ≥ 30 breaths/minute; oxygenation index (PaO_2_/FiO_2_) ≤ 250 mm Hg (1 mm Hg = 0.133 kPa); multilobar infiltrates; confusion and/or disorientation; blood urea nitrogen ≥ 7.14 mmol/L; systolic blood pressure < 90 mm Hg requiring aggressive fluid resuscitation.

### 2.2. Methods

Data collected included: demographics, clinical symptoms, past medical history, laboratory tests, chest CT imaging findings, antibiotic usage before and after diagnosis, and clinical outcomes. This study obtained informed consent from patients and was approved by the Ethics Committee of Hunan Second Provincial People’s Hospital.

The definition of coinfection was based on the following criteria^[13]^: high sequence reads of the co-pathogen in BALF; clinical and imaging features consistent with mixed infection; favorable response to targeted antimicrobial therapy.

Statistical analysis: all statistical analyses were performed using Statistical Package for the Social Sciences Statistics (version 26.0; International Business Machines Corporation). Given the small sample size (n = 10) and the descriptive nature of this study, no inferential statistical tests were applied. All analyses were purely descriptive, focusing on summarizing demographic, clinical, laboratory, imaging, and therapeutic characteristics of the cohort. Continuous variables were assessed for normality using the Shapiro–Wilk test. Normally distributed data were presented as mean ± standard deviation, while non-normally distributed data were expressed as median with range. Categorical variables were summarized as frequencies and percentages (%).

## 3. Results

### 3.1. Baseline characteristics

Among the 10 patients (6 males, 4 females), age ranged from 28 to 76 years (median: 70 years, mean: 63.3 ± 15.0 years). The disease course before admission ranged from 1 to 9 days (median: 4.5 days, mean: 4.3 ± 2.6 days). Hospital stay ranged from 6 to 10 days (mean: 9.6 ± 3.4 days). Nine patients had comorbidities: hypertension (n = 4), diabetes mellitus (n = 3), coronary heart disease (n = 2), and hepatitis B (n = 1). Four patients reported a history of bird contact before onset, while 6 had no clear exposure history (Table 1). To further explore the impact of underlying comorbidities on disease severity and outcomes, a descriptive subgroup analysis was performed (Table 2).

**Table 1:** Clinical characteristics of patients with *Chlamydia psittaci* pneumonia.

Case number	Gender	Age (yrs)	Disease course (d)	Hospital stay (d)	Underlying diseases	Poultry exposure	Severe pneumonia	Initial symptom	Peak temperature (°C)	Accompanying symptoms
Case 1	Female	61	4	8	Hypertension, Diabetes	Yes	No	Fever	40	Dry cough, dizziness, fatigue, myalgia
Case 2	Male	70	2	16	Coronary heart disease	No	Yes	Fever	39.8	Chills, fatigue, anorexia, dyspnea
Case 3	Male	70	5	14	Hypertension, Coronary heart disease	No	Yes	Fever	39	Chills, anorexia
Case 4	Male	48	3	8	Diabetes	No	No	Fever	39.5	Chills, rigors, headache, myalgia, nausea
Case 5	Female	28	10	7	None	No	No	Fever	39.6	Dry cough, tachypnea, headache, myalgia
Case 6	Female	76	6	8	Diabetes, Cerebral infarction	No	No	Fever	38.9	Cough
Case 7	Male	71	2	9	Cerebral infarction,	Yes	No	Fever	39	Chills, rigors, cough
Case 8	Male	76	1	13	Hypertension, Atrial fibrillation	No	No	Fever	39	Anorexia
Case 9	Male	62	5	7	Fatty liver, Hepatitis B	Yes	No	Fever	41	Cough, anorexia
Case 10	Female	71	5	6	Hypertension	Yes	No	Fever	39	Chills, rigors, headache, nausea, anorexia

**Table 2:** Descriptive subgroup analysis of clinical characteristics and outcomes according to underlying comorbidities.

Comorbidity	n	Severe pneumonia (n)	Hospital stay (d, mean ± SD)	Hyponatremia, n (%)	Favorable outcome, n (%)
Hypertension	4	1	10.3 ± 3.9	4 (100)	4 (100)
Diabetes	3	0	8.0 ± 0.0	2 (66.7)	3 (100)
Coronary heart disease	2	2	15.0 ± 1.4	2 (100)	2 (100)
Cerebral infarction	2	0	8.5 ± 0.7	2 (100)	2 (100)
No comorbidity	1	0	7	1 (100)	1 (100)

Due to the small sample size, no statistical comparisons were performed between subgroups. Data are presented descriptively only. Serum sodium level **<** 135 mmol/L is defined as hyponatremia.

n = number of patients, SD = standard deviation.

### 3.2. Clinical manifestations

All patients presented with fever, with peak temperatures ranging from 38.9°C to 41.0°C (mean: 39.5 ± 0.7°C). Accompanying symptoms included cough (n = 4), chills (n = 5), rigors (n = 3), anorexia (n = 5), fatigue (n = 2), myalgia (n = 3), headache (n = 3), nausea (n = 2), and dyspnea (n = 1). Two patients with severe pneumonia developed neurological symptoms such as delirium (Table 1).

### 3.3. Laboratory findings

Laboratory results on admission day: 8 patients met the criteria for type I respiratory failure (PaO_2_/FiO_2_ ≤ 300 mm Hg), one patient had mildly reduced oxygenation (PaO_2_/FiO_2_ between 300 and 400 mm Hg) without meeting the criteria for respiratory failure, and only one patient had normal oxygenation (PaO_2_/FiO_2_ > 400 mm Hg). The white blood cell (WBC) count was normal in 9 patients, and slightly elevated in 1. The neutrophil count was mildly elevated in 6 patients. The lymphocyte count was decreased in 9 patients. C-reactive protein was elevated in all patients, with levels > 200 mg/L in 4. Procalcitonin and interleukin-6 were elevated in 9 patients. Alanine aminotransferase and aspartate aminotransferase were elevated in 9 patients. Hyponatremia (serum sodium level < 135 mmol/L) occurred in 9 patients. The 2 patients with severe pneumonia developed rhabdomyolysis, with markedly elevated creatine kinase and myoglobin levels (Table 3).

**Table 3:** Laboratory findings of patients with *Chlamydia psittaci* pneumonia.

Case number	PaO_2_/FiO_2_ (mm Hg)	WBC (×10^9^/L)	NEUT (×10^9^/L)	LYMPH (×10^9^/L)	CRP (mg/L)	PCT (ng/mL)	IL-6 (pg/mL)	ALT (U/L)	AST (U/L)	CK (U/L)	Myoglobin (μg/L)	Serum Na^+^ (mmol/L)
Case 1	296	8.81	8.07	0.5	306.3	0.367	214.9	85.8	129.3	136.3	65	132
Case 2	121	5.76	5.33	0.34	270.1	4.44	453.6	83	43.8	1956.5	255	131
Case 3	214	9.37	7.99	0.68	212.3	0.7	282.4	41.5	47	508.3	383.9	130
Case 4	423	3.1	2	0.77	7.2	<0.02	<1.5	53.5	54.6	34.1	ND	138
Case 5	283	5.27	3.76	1.02	137.9	0.133	110.6	51.6	20.1	37.6	34	123
Case 6	213	12.71	10.51	0.9	179	0.166	46.1	96.1	77	72.1	27.4	132
Case 7	242	8.94	7.4	0.63	111.2	0.41	222.8	70	60	126.6	69.6	133
Case 8	382	9	6.83	1.35	80.8	0.094	120	17.2	10.7	164	95.6	131
Case 9	286	7.85	6.93	0.49	330	0.47	423	70.4	64	77	22	131
Case 10	233	5.83	4.9	0.69	170.7	0.477	84.5	68.3	42.8	ND	ND	129

ALT = alanine aminotransferase, AST = aspartate aminotransferase, CK = creatine kinase, Cl^−^ = chloride ion, CRP = C-reactive protein, IL-6 = interleukin-6, K^+^ = potassium ion, LYMPH = lymphocyte count, Myo = myoglobin, Na^+^ = sodium ion, ND = not detected, NEUT = neutrophil count, PaO_2_/FiO_2_ = partial pressure of arterial oxygen/fraction of inspired oxygen, PCT = procalcitonin, WBC = white blood cell.

### 3.4. Imaging Findings

Chest CT revealed consolidation with air bronchograms in 5 patients; mass-like lesions in 2; and ground-glass opacities (GGO) in 3. All patients had unilateral lung involvement. Nine patients had single-lobe involvement; 1 involved both the right middle and lower lobes. Lesions were predominantly in the lower lobes (left lower lobe: n = 3; right lower lobe: n = 4). Pleural involvement was seen in 9 cases, with pleural effusion in 2 (Table 4, Fig. 1).

**Table 4:** Imaging features of patients with *Chlamydia psittaci* pneumonia.

Case number	Location	Pattern	Air bronchogram	Pleural effusion	Pleural involvement
Case 1	RLL	Consolidation	Yes	No	Yes
Case 2	LLL	Consolidation	Yes	Yes	Yes
Case 3	RLL	Consolidation	Yes	No	Yes
Case 4	LLL	Mass	Yes	No	Yes
Case 5	LUL	Ground-glass opacity	Yes	No	Yes
Case 6	RUL	Ground-glass opacity	Yes	No	Yes
Case 7	LLL	Consolidation	Yes	No	Yes
Case 8	RUL	Mass	Yes	No	No
Case 9	RML, RLL	Consolidation	Yes	Yes	Yes
Case 10	RLL	Ground-glass opacity	No	No	Yes

LLL = left lower lobe, LUL = left upper lobe, RLL = right lower lobe, RML = right middle lobe, RUL = right upper lobe.

**Figure 1.**
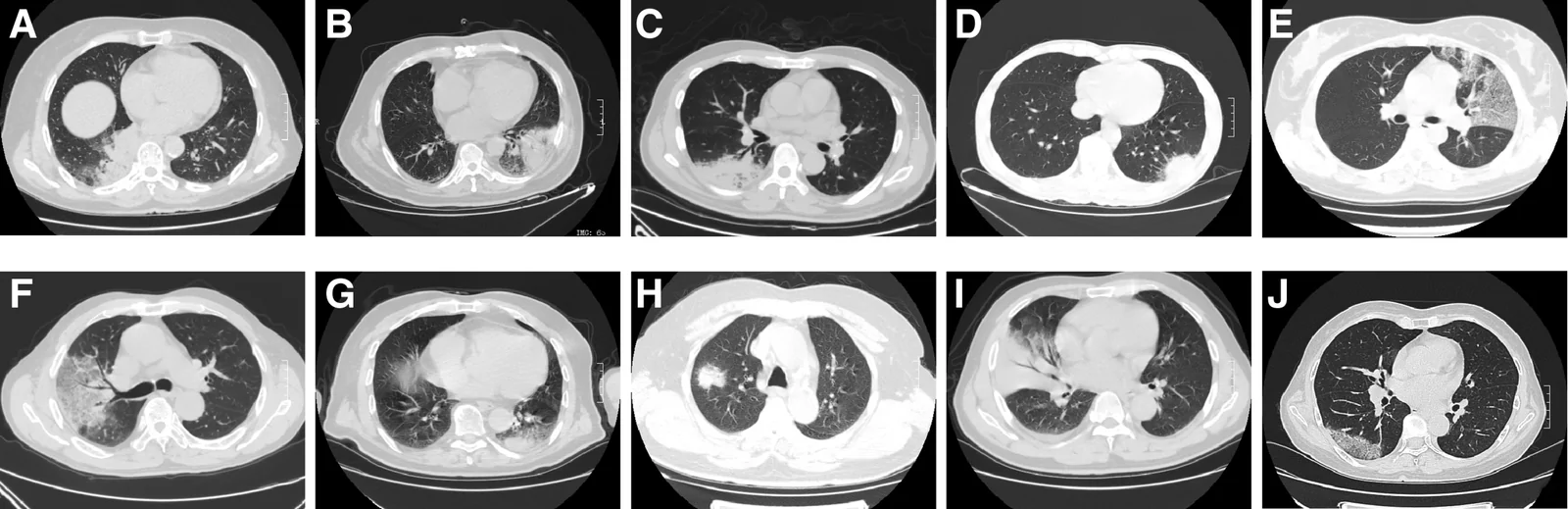
Chest CT imaging findings of 10 patients in this study. CT = computed tomography.

### 3.5. mNGS and tNGS Results

Five patients underwent BALF mNGS, and 5 underwent tNGS; *C psittaci* sequences were detected in all. Co-detected organisms (n = 5) included: *Staphylococcus aureus*, *Haemophilus influenzae*, *Serratia marcescens*, *Klebsiella pneumoniae*, *Stenotrophomonas maltophilia*, *Parvimonas micra*, *Fusobacterium nucleatum*, *Candida albicans*, Epstein–Barr virus, and herpes simplex virus type 1 (HSV-1). Only *S aureus* and *H influenzae* were judged as true co-infections; others were likely colonization or transient carriage (Table 5).

**Table 5:** Next-generation sequencing results of bronchoalveolar lavage fluid in *Chlamydia psittaci* pneumonia.

Case number	Sample type	Detection method	*Chlamydia psittac* sequence count	Co-detected pathogens (sequence count)
Case 1	BALF	mNGS	3862	*Parvimonas micra* (132), EBV (30)
Case 2	BALF	mNGS	12,932	-
Case 3	BALF	mNGS	921	-
Case 4	BALF	mNGS	3094	-
Case 5	BALF	mNGS	216	-
Case 6	BALF	tNGS	1669	HSV-1 (1236); *Candida albicans* (20,492); *Fusobacterium nucleatum* (174)
Case 7	BALF	tNGS	1619	Staphylococcus aureus* (4155), Epstein–Barr virus (201)
Case 8	BALF	tNGS	2290	Haemophilus influenzae* (3379); *Serratia marcescens* (51,244); *Klebsiella pneumoniae* (43)
Case 9	BALF	tNGS	11,834	-
Case 10	BALF	tNGS	8083	*Stenotrophomonas maltophilia* (97)

BALF = bronchoalveolar lavage fluid, EBV = Epstein–Barr virus, mNGS = metagenomic next-generation sequencing, tNGS = targeted next-generation sequencing.

Pathogens with an asterisk (*) in the “co-detected pathogens” column were classified as true infection in this study; those without an asterisk were considered colonization or carriage.

Although a formal comparative analysis of cost and turnaround time was not the primary endpoint, institutional data revealed that the approximate cost of tNGS was one-sixth that of mNGS, with an average turnaround time of 18 to 24 hours for tNGS compared to 48 to 60 hours for mNGS in our center. Both methods demonstrated 100% detection concordance in this small cohort, but further head-to-head studies are needed to definitively compare sensitivity and specificity.

### 3.6. Treatment and outcomes

Before admission, most patients (n = 8) had received empirical β-lactam antibiotics (e.g., amoxicillin, ceftazidime, cefixime). After admission but prior to diagnosis, 5 patients received empirical moxifloxacin or doxycycline. Of the other 5, 4 initially received piperacillin-tazobactam, and 1 received meropenem. Due to lack of improvement, all 5 were switched to moxifloxacin or a tetracycline (doxycycline or omadacycline) before diagnosis confirmation. As these regimens were effective, they were continued post-diagnosis. One severe case received doxycycline plus moxifloxacin post-diagnosis for enhanced efficacy and coverage of *S aureus*. Post-discharge, 1 patient on moxifloxacin was switched to oral doxycycline, and 1 to oral nemonoxacin. Treatment duration was 10 to 21 days. All patients improved with good prognoses (Table 6).

**Table 6:** Therapeutic regimens and outcomes in *Chlamydia psittaci* pneumonia.

Case number	Preadmission empirical therapy	In-hospital empirical therapy	Targeted therapy	Treatment duration (d)	Outcome
Case 1	Cefuroxime	Moxifloxacin	Moxifloxacin	16	Improved
Case 2	-	Meropenem + Doxycycline	Doxycycline + Moxifloxacin	14	Improved
Case 3	Ceftazidime	Piperacillin-tazobactam + Moxifloxacin	Moxifloxacin	13	Improved
Case 4	Cefixime	Doxycycline	Doxycycline	21	Improved
Case 5	Cephalosporin	Moxifloxacin	Moxifloxacin → Doxycycline	14	Improved
Case 6	Cephalosporin	Piperacillin-tazobactam + Doxycycline	Doxycycline	14	Improved
Case 7	-	Piperacillin-tazobactam + Moxifloxacin	Moxifloxacin → Nemonoxacin	15	Improved
Case 8	Amoxicillin	Piperacillin-tazobactam + Omadacycline	Omadacycline	10	Improved
Case 9	Cefuroxime	Moxifloxacin	Moxifloxacin	21	Improved
Case 10	Ceftriaxone	Moxifloxacin	Moxifloxacin	14	Improved

## 4. Discussion

*C psittaci* is a gram-negative, obligate intracellular pathogen. It can infect mammals, including humans, causing severe psittacosis. Delayed diagnosis and consequently delayed initiation of effective antibiotics can lead to high mortality in severe cases.^[14]^ Due to diagnostic limitations, the incidence of *C psittaci* pneumonia is chronically underestimated; nationwide epidemiological data in China are lacking.^[2]^

Only 40% of our patients had a bird contact history, consistent with previous cohort studies,^[2,7]^ suggesting insidious onset. Human infection typically occurs via inhalation of dust/aerosols from dried bird feces, urine, feathers, or respiratory secretions, or through contact with infected secretions, blood, or tissues (e.g., during slaughter, plucking) via broken skin/mucosa. Rare human-to-human transmission has occurred.^[15]^ In densely populated, highly mobile societies, indirect exposure is likely; the absence of a clear contact history cannot rule out psittacosis. Clinical presentation is nonspecific, often featuring high fever, chills, headache, myalgia, and fatigue, with or without respiratory symptoms.^[16]^ All 10 patients presented with moderate-to-high fever; respiratory symptoms were mild, while systemic manifestations (chills, rigors, myalgia, fatigue) were common. Limited clinician awareness contributes to misdiagnosis. While progression to severe pneumonia occurs, most cases are curable.^[10,17,18]^

Arterial blood gas analysis revealed type I respiratory failure in 8 patients, while 1 additional patient had mildly reduced oxygenation (PaO_2_/FiO_2_ between 300 and 400 mm Hg) without meeting the criteria for respiratory failure; only 1 had normal oxygenation, underscoring the need for vigilant oxygenation monitoring in psittacosis. While WBC and neutrophil counts were normal or mildly elevated, lymphocytes were decreased. Marked elevations in C-reactive protein, procalcitonin, and interleukin-6 were consistent with prior studies,^[2,11]^ indicating a rapid acute-phase response protein rise despite minimal early WBC elevation. Elevated liver enzymes (n = 9) occur as *C psittaci* initially proliferates in the reticuloendothelial cells of the liver and spleen after inhalation.^[9]^ Hyponatremia, rarely emphasized previously and reported in only 2 cases,^[19]^ was highly prevalent (90%) in our cohort. Chlamydia, along with *Mycoplasma* and *Legionella pneumophila*, belongs to atypical pathogens. The incidence of hyponatremia in individuals infected with *Legionella* reaches 38 to 67%.^[20,21]^ Hyponatremia increases mortality, prolongs hospitalization, and is an independent predictor of severity and intensive care unit admission.^[22]^ The high rate of hyponatremia here underscores the need to monitor sodium levels in psittacosis patients. The mechanism for hyponatremia in atypical pneumonia remains unclear, potentially involving the syndrome of inappropriate antidiuretic hormone secretion or direct renal salt loss due to endotoxins/cytokines.^[23,24]^

*C psittaci* pneumonia imaging typically shows lesions distributed along secondary pulmonary lobules, peribronchovascular bundles, subpleural areas, or diffusely presenting as consolidation and GGO,^[8]^ but lacking necrotic cavities or tree-in-bud signs. Our cases mainly showed GGO, consolidation, or mass-like lesions without necrosis, mostly with pleural involvement (n = 9), which is rarely reported in other studies.

Laboratory diagnosis of *C psittaci* includes culture, serology, and polymerase chain reaction (PCR). Culture is time-consuming and requires Biosafety Level 3 facilities.^[25]^ Serology and PCR lack sufficient sensitivity and specificity for routine clinical use. Recently, NGS has significantly improved diagnostic capabilities. In our cohort, 5 patients were diagnosed by mNGS and 5 by tNGS, respectively. mNGS allows unbiased detection of diverse pathogens (bacteria, fungi, viruses, parasites). Despite improved detection, mNGS faces challenges like high cost, insufficient sequencing depth, and complex workflows.^[26]^ tNGS combines ultra-multiplex PCR amplification/probe capture with NGS, enabling simultaneous DNA/RNA analysis for dozens to hundreds of known pathogens.^[27]^ It offers faster turnaround and better cost-saving advantages, though its application for *C psittaci* detection is less studied.^[11]^ Our study indicated that when psittacosis is suspected clinically, tNGS may offer a viable option to reduce disease burden, shorten time-to-diagnosis, and conserve medical resources.

All patients achieved good outcomes due to timely diagnosis and treatment. *C psittaci* lacks a typical cell wall, conferring intrinsic resistance to cell-wall targeting antibiotics (e.g., β-lactams). It is susceptible to antibiotics interfering with DNA/protein synthesis: tetracyclines, macrolides, and fluoroquinolones. Doxycycline, with its rapid penetration, high cure rate, and mild side effects, is often first-line for severe psittacosis.^[28]^ Most patients in our study received ineffective penicillins, cephalosporins, or even carbapenems empirically before admission, highlighting diagnostic challenges. Although the 2 severe cases had longer hospital stays, prompt switch to fluoroquinolones or tetracyclines before definitive diagnosis, guided by clinical suspicion, led to good outcomes without significant adverse effects. Early identification and correct management of potentially severe cases are crucial.

We acknowledge several limitations. First, the small sample size (n = 10) limits the generalizability of our findings, particularly the precise estimation of prevalence for features like hyponatremia. Hyponatremia was highly prevalent in this cohort of patients with *C psittaci* pneumonia (90% in our cohort), although this finding may not be specific to psittacosis and warrants further investigation in milder cases. Second, the small cohort precludes meaningful subgroup analyses or multivariate regression to identify independent predictors of severity or treatment response. Furthermore, a quantitative assessment of pathogen load was not performed in this study, precluding independent validation of our microbiological classification via conventional microbiology (e.g., culture or PCR).

## 5. Conclusion

*C psittaci* pneumonia primarily presents with fever and systemic symptoms, with relatively mild respiratory manifestations. Most patients have normal WBC, decreased lymphocytes, but markedly elevated acute-phase proteins. Elevations in liver enzymes, impaired oxygenation, and hyponatremia are highly prevalent in this cohort of patients with *C psittaci* pneumonia. Chest CT typically shows consolidation, GGO, or mass-like lesions with air bronchogram, often with pleural involvement. Both tNGS and mNGS are effective for etiological diagnosis, with tNGS offering potential advantages in cost and turnaround time. Early diagnosis and treatment with fluoroquinolones (e.g., moxifloxacin) or tetracyclines (e.g., doxycycline, omadacycline) lead to favorable outcomes.

## Author contributions

**Conceptualization:** Rongchun Wang, Xiangbo Zeng.

**Data curation:** Rongchun Wang, Na Li, Xiangbo Zeng.

**Investigation:** Jia Tian.

**Methodology:** Rongchun Wang, Xiangbo Zeng.

**Project administration:** Rongchun Wang.

**Writing – original draft:** Rongchun Wang.

**Writing – review & editing:** Rongchun Wang, Na Li, Xiangbo Zeng.

## References

[1] Broderick DTJ Regtien T Ainsworth A Taylor MW Pillarisetti N. Dynamic upper and lower airway microbiotas in paediatric bronchiectasis exacerbations: a pilot study. Front Cell Infect Microbiol. 2022;11:773496. 10.3389/fcimb.2021.773496 PMC8818954 35141165

[2] Yang M Yang DH Yang H. Clinical characteristics of chlamydia psittaci pneumonia infection in Central South China. Infect Dis Ther. 2022;11:1631–47. 10.1007/s40121-022-00662-4 PMC9207437 35723864

[3] Hogerwerf L De Gier B Baan B Van Der Hoek W. Chlamydia psittaci (psittacosis) as a cause of community-acquired pneumonia: a systematic review and meta-analysis. Epidemiol Infect. 2017;145:3096–105. 10.1017/S0950268817002060 PMC9148753 28946931

[4] Qu J Zhang J Chen Y. Aetiology of severe community acquired pneumonia in adults identified by combined detection methods: a multi-centre prospective study in China. Emerg Microbes Infect. 2022;11:556–66. 10.1080/22221751.2022.2035194 PMC8843176 35081880

[5] Yin Q Li Y Pan H. Atypical pneumonia caused by Chlamydia psittaci during the COVID-19 pandemic. Int J Infect Dis. 2022;122:622–7. 10.1016/j.ijid.2022.07.027 PMC9276535 35842216

[6] de Gier B Hogerwerf L Dijkstra F van der Hoek W. Disease burden of psittacosis in the Netherlands. Epidemiol Infect. 2018;146:303–5. 10.1017/S0950268817003065 PMC9134569 29361998

[7] Deng H Shi Y Xie M. Diagnosis and treatment experience of Chlamydia psittaci pneumonia: a multicenter retrospective study in China. BMC Infect Dis. 2024;24:1333. 10.1186/s12879-024-10198-2 PMC11583410 39578769

[8] Wu J Pan J Han C. Clinical and CT diagnosis of 50 cases of Chlamydia psittaci pneumonia. Quant Imaging Med Surg. 2023;13:2053–64. 10.21037/qims-22-809 PMC10102789 37064368

[9] Yang F Li J Qi B. Clinical symptoms and outcomes of severe pneumonia caused by Chlamydia psittaci in Southwest China. Front Cell Infect Microbiol. 2021;11:727594. 10.3389/fcimb.2021.727594 PMC8770948 35071027

[10] Chen X Cao K Wei Y. Metagenomic next-generation sequencing in the diagnosis of severe pneumonias caused by Chlamydia psittaci. Infection. 2020;48:535–42. 10.1007/s15010-020-01429-0 PMC7223968 32314307

[11] Zhang Y Jiang X Ye W Sun J. Clinical features and outcome of eight patients with Chlamydia psittaci pneumonia diagnosed by targeted next generation sequencing. Clin Respir J. 2023;17:915–30. 10.1111/crj.13681 PMC10500319 37552996

[12] Metlay JP Waterer GW Long AC. Diagnosis and treatment of adults with community-acquired pneumonia. an official clinical practice guideline of the American Thoracic Society and Infectious Diseases Society of America. Am J Respir Crit Care Med. 2019;200:e45–67. 10.1164/rccm.201908-1581ST PMC6812437 31573350

[13] Chinese Thoracic Society. Consensus of clinical pathways of metagenomic next-generation sequencing test in diagnosis of lower respiratory tract infections in China. Zhonghua Jie He He Hu Xi Za Zhi. 2023;46:322–35. 10.3760/cma.j.cn112147-20220701-00553 36787941

[14] Shi Y Chen J Shi X. A case of chlamydia psittaci caused severe pneumonia and meningitis diagnosed by metagenome next-generation sequencing and clinical analysis: a case report and literature review. BMC Infect Dis. 2021;21:621. 10.1186/s12879-021-06205-5 PMC8243071 34193063

[15] Zhang Z Zhou H Cao H. Human-to-human transmission of Chlamydia psittaci in China, 2020: an epidemiological and aetiological investigation. Lancet Microbe. 2022;3:e512–20. 10.1016/S2666-5247(22)00064-7 35617977

[16] Knittler MR Sachse K. Chlamydia psittaci: update on an underestimated zoonotic agent. Pathog Dis. 2015;73:1–15. 10.1093/femspd/ftu007 25853998

[17] Gu L Liu W Ru M. The application of metagenomic next-generation sequencing in diagnosing Chlamydia psittaci pneumonia: a report of five cases. BMC Pulm Med. 2020;20:65. 10.1186/s12890-020-1098-x PMC7077129 32178660

[18] Dai N Li Q Geng J Guo W Yan W. Severe pneumonia caused by Chlamydia psittaci: report of two cases and literature review. J Infect Dev Ctries. 2022;16:1101–12. 10.3855/jidc.16166 35797307

[19] Heqiang L Xiu P Xiaohong W. Two cases of Chlamydia psittaci pneumonia and literature review. China Modern Med. 2024;31:140–5.

[20] Weixuan L Yuanjue Z Weici L Bixiang Z. Analysis of 37cases of penumonia of Legionnaires’ disease. Chin J Tuberc Respir Dis. 1997;22:91–7.

[21] Yu VL. Legionnaires’ disease: importance of high index of suspicion in patients in the ICU with community-acquired pneumonia. Chest. 2014;145:202–5. 10.1378/chest.13-2170 24493498

[22] Arancibia F Cortes CP Valdes M. Importance of Legionella pneumophila in the etiology of severe community-acquired pneumonia in Santiago, Chile. Chest. 2014;145:290–6. 10.1378/chest.13-0162 23764871

[23] Schuetz P Haubitz S Christ-Crain M Albrich WC Zimmerli W Mueller B; ProHOSP Study Group. Hyponatremia and anti-diuretic hormone in Legionnaires’ disease. BMC Infect Dis. 2013;13:585. 10.1186/1471-2334-13-585 PMC3880094 24330484

[24] Eisenhut M. Changes in ion transport in inflammatory disease. J Inflamm (Lond). 2006;3:5. 10.1186/1476-9255-3-5 PMC1562419 16571116

[25] Balsamo G Maxted AM Midla JW. Compendium of measures to control Chlamydia psittaci infection among humans (psittacosis) and pet birds (avian chlamydiosis), 2017. J Avian Med Surg. 2017;31:262–82. 10.1647/217-265 28891690

[26] Chinese Society of Laboratory Medicine. Expert consensus on clinical standardized application of metagenomics next‐generation sequencing for detection of pathogenic microorganisms. Chin J Lab Med. 2020;43:1181–95.

[27] The Committee Of Chinese Laboratory Medical Education CTS. Expert consensus on nucleic acid of amplification techniques test of respiratory tract infection in adults (2023). Med J Peking Union Med Coll Hosp. 2023;14:959–71.

[28] Hammerschlag MR Kohlhoff SA. Treatment of chlamydial infections. Expert Opin Pharmacother. 2012;13:545–52. 10.1517/14656566.2012.658776 22316336

